# A novel heterozygous mutation of *ANKRD11* causes KBG syndrome in a preterm neonate: a case report and literature review

**DOI:** 10.3389/fped.2025.1565261

**Published:** 2025-06-12

**Authors:** Jie Gao, Ruiqin Wang, Zhen Pan, Ruolan Hu, Mingyan Jiang, Jinrong Li

**Affiliations:** ^1^ Department of Pediatrics, West China Second University Hospital, Sichuan University, Chengdu, Sichuan, China; ^2^ Key Laboratory of Birth Defects and Related Diseases of Women and Children, Ministry of Education, Sichuan University, Chengdu, Sichuan, China; ^3^ Medical College, Mudanjiang Medical University, Mudanjiang, Heilongjiang, China; ^4^ West China School of Medicine, Sichuan University, Chengdu, Sichuan, China

**Keywords:** *ANKRD11*, congenital chylothorax, KBG syndrome, premature, heterozygous mutation

## Abstract

The KBG syndrome (KBGS) affects several systems caused by the mutation of the *ANKRD11* gene. The main manifestations of KGBS included hearing loss, feeding difficulties, craniofacial abnormalities, tooth deformity, and developmental delay (delayed overall development, convulsions, and intellectual abnormalities). Only 10%–26% of patients with KBG syndrome have congenital heart disease, including atrial and ventricular septal defects. Here, we report a case of KBG syndrome in a preterm newborn with low birth weight, a huge ventricular septal defect, and a congenital chylothorax. Whole-exome sequencing detected an *ANKRD11* gene mutation in the infant. The finding expands the understanding of the clinical and genetic phenotype. The multidisciplinary consultation of the complex KGB syndrome including interventional occlusion, nutritional management, and rehabilitation training can improve the prognosis and outcome.

## Introduction

The genesis of KBG syndrome (OMIM 148050) involves either heterozygous mutations within the ankyrin repeat domain-containing protein 11 (*ANKRD11*) or the deletions in the 16q24.3 region that include the *ANKRD11* gene (1). This syndrome was characterized by distinctive facial features, developmental delay, short stature, and skeletal anomalies (2). Despite existing diagnostic criteria, some patients exhibited subtle clinical features, and no single trait was indispensable for diagnosis, leading to misdiagnosis (3). However, advances in whole-exome sequencing have expanded our understanding of genetic variations related to KBG syndrome and highlighted the complexity of its phenotypic presentation (4).

Within the context of genetic research and clinical practice, the identification of novel phenotypes contributes to a more comprehensive genetic and clinical database. This facilitated the refinement of genetic testing panels for KBG syndrome, improving diagnostic accuracy and potentially identifying novel therapeutic targets. We reported a groundbreaking case of a preterm neonate with congenital chylothorax, verified through whole-exome sequencing to result from a novel heterozygous mutation in *ANKRD11*. This case broadens the recognized phenotypic and genetic spectrum of KBG syndrome, contributing to the understanding of the syndrome's genetic diversity. It underscored the challenges of diagnosing and managing KBG syndrome in preterm infants with comorbid conditions, highlighting the pivotal role of genetic testing in these complex scenarios.

## Case report

The prohand was admitted to our hospital due to premature birth and cyanosis at birth. She was born a non-consanguineous couple by cesarean surgery at 36 + 3 weeks of gestation while the birth weight was 2,470 g and the height was 49 cm. The Apgar scores at 1–5–10 min were 5 (respiration, 2 points; heart rate, muscle tone, skin color 1 point), 9 (skin color, 1 point), and 10 points, respectively.

**Figure 1:**
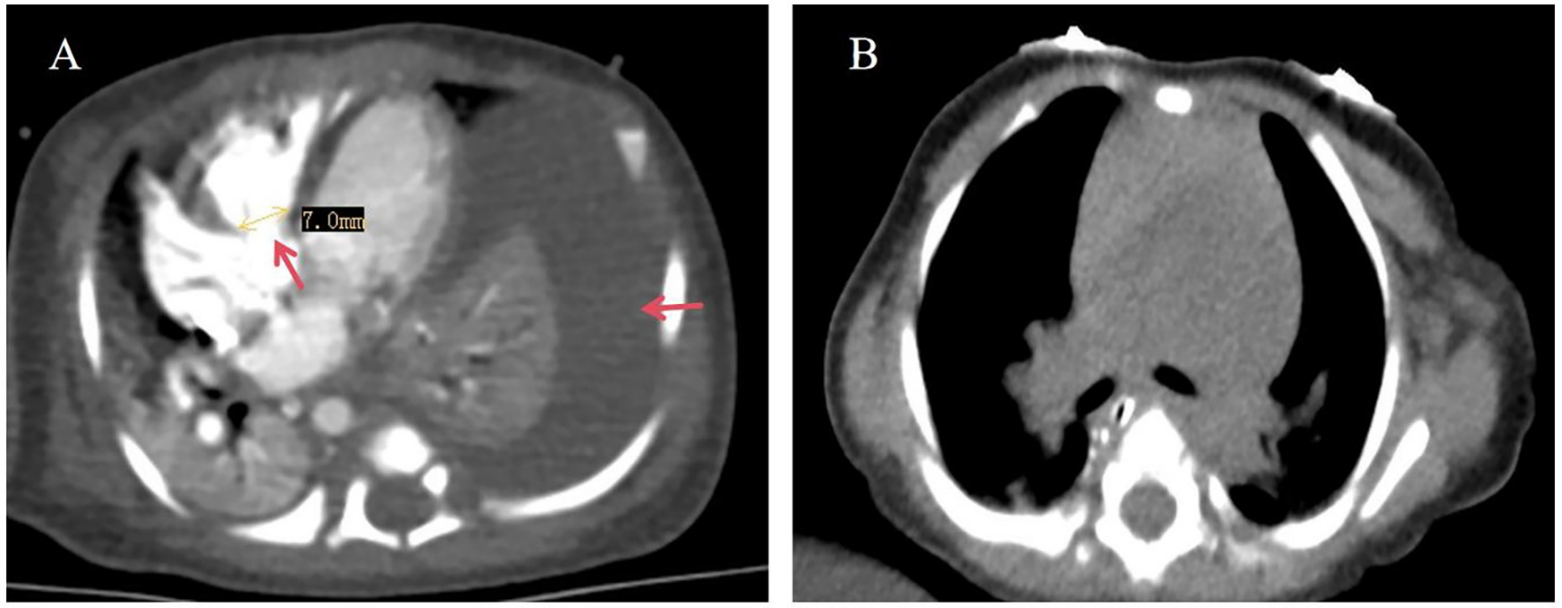
**(A)** Ventricular septal defect, large left pleural effusion before celiac chest sealing; **(B)** sealing after chest radiograph.

Pleural effusion analysis revealed a lymphocyte count of 97.0%, a nucleated cell count of 36,422 × 10^6^g/L, and positive pleural fluid protein by qualitative testing. Chest radiograph revealed a left-sided pleural effusion, and echocardiography showed a mixed-type ventricular septal defect, hypoplastic aortic arch (AO = 7 mm, sinus junction ∼6 mm, AAO = 7 mm, transverse arch 5 mm, isthmus 3.5 mm, and descending aorta ∼4.5 mm) and other cardiac anomalies (Figure 1A).

**Figure 2:**
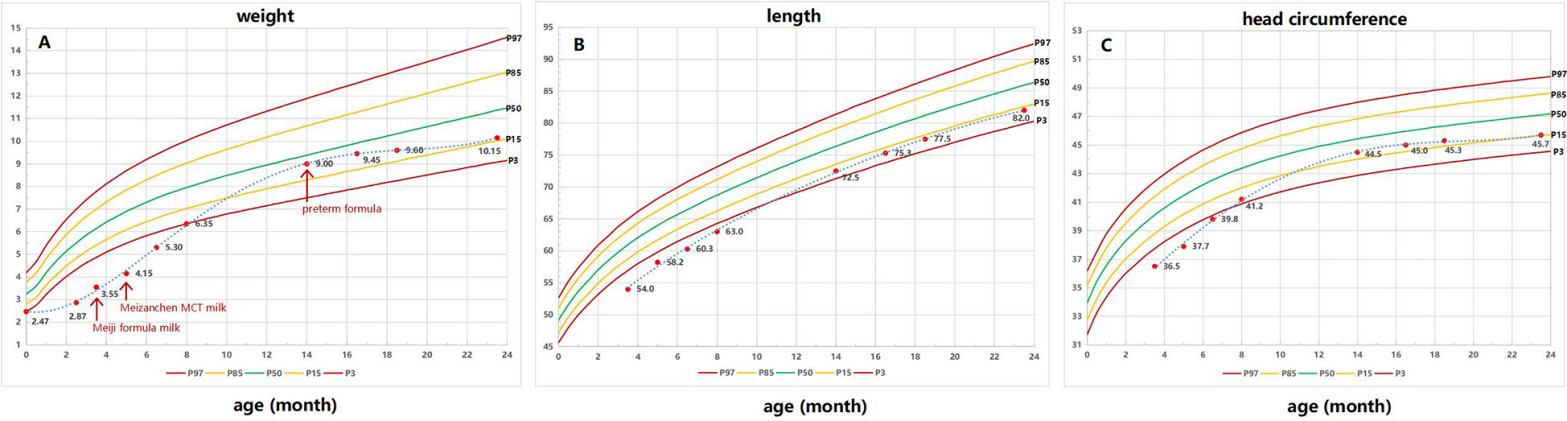
Growth chart of the patient. The triangle indicates the physical measurements. The arrow marks the beginning of Meiji formula milk/Meizanchen MCT milk/preterm formula. **(A)** Weight growth curve; **(B)** height growth curve; **(C)** head circumference growth curve.

After birth, the prohand received ventilator support, somatostatin infusion, continuous closed thoracic drainage, and special formula feeding with medium-chain triglycerides (MCT; 100 ml supplying 3.42 g MCT, 70 kcal), at 400–600 ml/day, supervised by the clinical nutrition team. Due to persistent poor growth, the feeding was switched to Meizanchen MCT formula (87% MCT, 100 kcal/100 ml). Four-month follow-up chest CT showed persistent chylothorax, prompting interventional occlusion surgery, which led to full resolution of the effusion (Figure 1B). To promote catch-up growth and support recovery, feeding was subsequently transitioned to high-caloric preterm formula (100 kcal/100 ml) (Figure 2). Surgical closure of the ventricular septal defect was performed at 10 months of age. The patient and her family demonstrated good compliance and tolerability to all interventions, including specialized nutritional formulas, surgical treatment, and rehabilitation training. Regular and timely pediatric follow-ups were maintained, and no significant adverse reactions or intolerance to treatments were observed.

**Figure 3:**
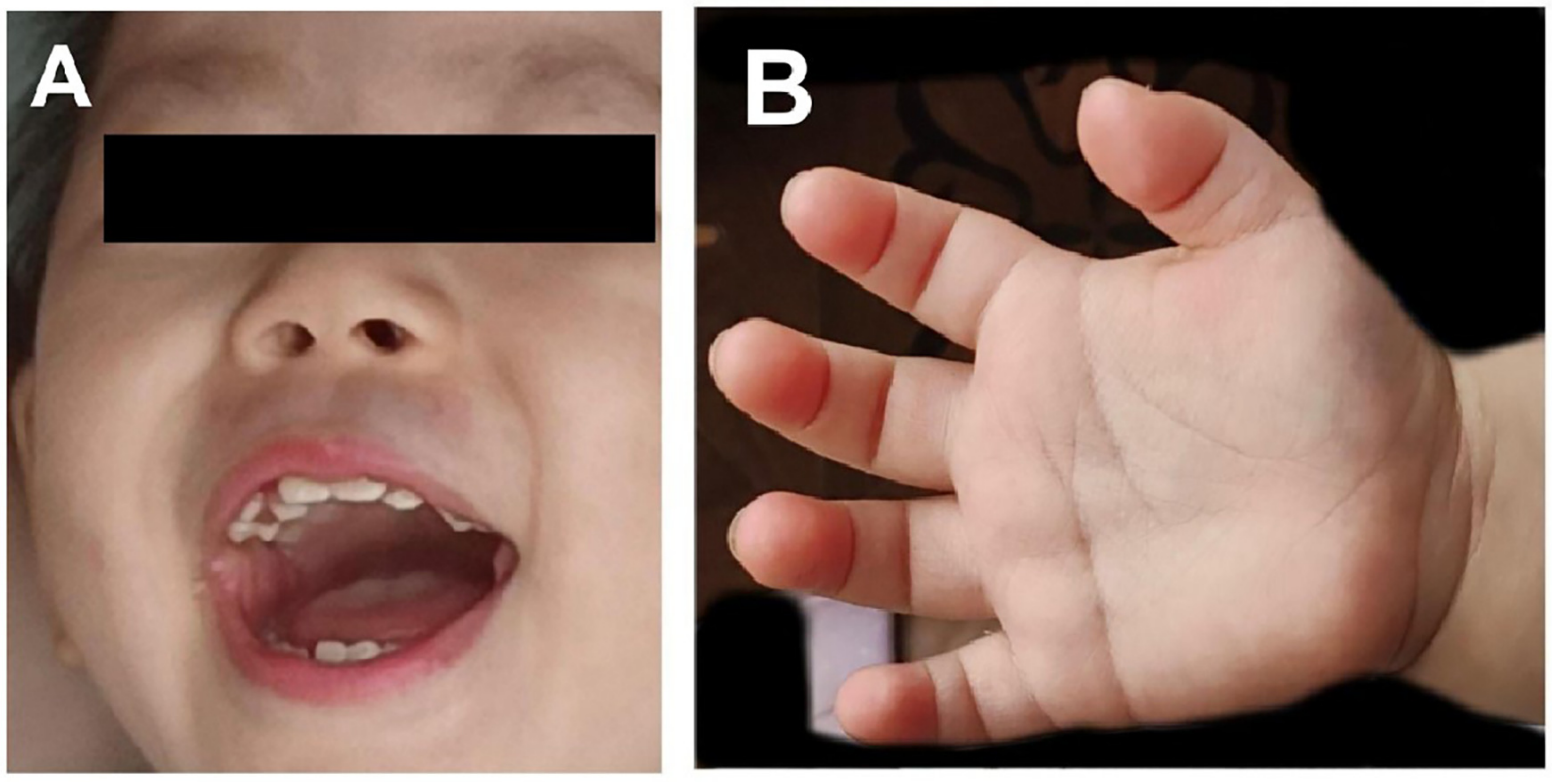
Clinical evaluation of individuals with *ANKRD11* missense variants. **(A)** Facial features, including larger and broader central incisors; **(B)** shortened fingers.

At 23 months old, the weight was 10.15 kg (P10–P20), length was 82 cm (P10–P20), and head circumference was 45.7 cm (P10–P20). The proband had larger and broader central incisors, shorter fingers, and larger, low-set ears and was also accompanied by mild torticollis. There was no anomaly in the abdomen or lungs, and there was a mild murmur (Level I) in the precordial area. There were no anomalies in the external genitalia, the limbs' muscle tension was mildly reduced, and strength was normal (Figure 3).

Developmental milestones: she could raise her head at 4 months old, sit without help at 15 months old, stand with assistance at 17 months old, and was unable to walk alone at 18 months old. She could laugh out loud at 4 months old and speak “mum” at 17 months old, but still she was unable to describe any more than two words or short sentences and received rehabilitation training.

The examination with the Griffiths Mental Development Scales indicated a severe delay in gross motor skills and visual performance and language and a moderate delay in hand–eye coordination and social interaction. Bilateral ventricular widening, decreased paraventricular white matter, a broadened temporal angle, a diminished volume of the hippocampus and parahippocampal gyrus, and expanded cerebral sulci and fissures in both hemispheres were all observed on head magnetic resonance imaging (MRI). A minor hearing loss is revealed by the hearing test.

**Figure 4:**
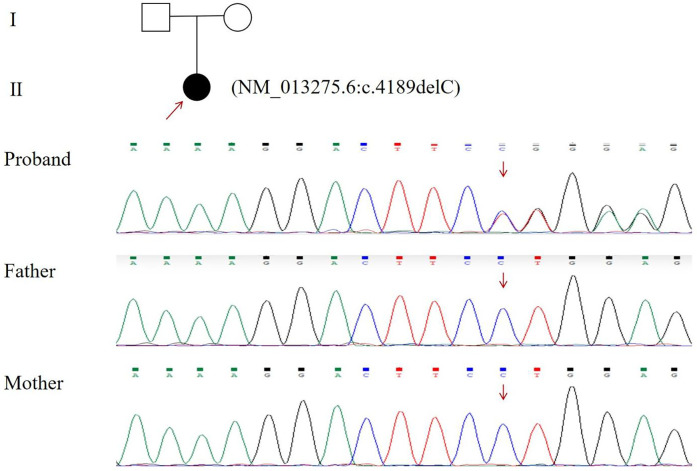
Sanger sequencing results of identified *ANKRD11* mutations. Affected residues were indicated with arrows.

Peripheral blood samples were collected from the patients and their parents. We performed next-generation sequencing (NGS) and used Sanger sequencing to confirm the results. Finally, we identified a variant in the *ANKRD11* gene (NM_013275.6:c.4189delC) (Figure 4). Following the ACMG guideline (5), the c.4189delC variant in ANKRD11 meets PVS1 (null variant in a gene with a loss-of-function mechanism), PM2 (absent in population databases), and PS2 (*de novo* occurrence confirmed by parental testing), supporting a pathogenic classification.

## Discussion

KBG syndrome is primarily diagnosed based on characteristic clinical features in combination with molecular confirmation of pathogenic variants in the *ANKRD11* gene or deletions involving the 16q24.3 region that includes *ANKRD11* (4, 6). However, no universally accepted clinical diagnostic criteria exist to date. Due to the broad phenotypic variability and overlapping features with other neurodevelopmental disorders, diagnosis can be particularly challenging. Choi et al. discovered that 62.5% of patients with KBG syndrome had cardiac abnormalities, such as ventricular septal defect, patent ductus arteriosus, and atrial septal defect (7, 8). Hearing impairment affected 27% of patients in the previously documented (9). Based on the literature, Low et al. (10) proposed a set of diagnostic criteria to assist clinicians in identifying KBG syndrome and categorizing features into major and minor groups. A summary of symptom prevalence based on their cohort is shown in Supplementary Figure S1, which provides a useful reference for evaluating clinical presentations (Supplementary Figure S1). According to these criteria, our patient met three of the four major features, including macrodontia of the maxillary central incisors, short stature, and hearing loss. Although the patient had no affected first-degree relatives, the *ANKRD11* variant was confirmed as *de novo*, adding to its pathogenic relevance. In addition, several minor features were present, such as short fingers, feeding difficulties during infancy, global developmental delay (in gross motor, language, and cognitive domains), and generalized hypotonia. These findings, in conjunction with the genetic evidence, strongly support the diagnosis of KBG syndrome in line with the clinical features observed in our patient corresponding well with several of the most prevalent symptoms reported in that dataset, further substantiating the diagnosis.

A particularly noteworthy feature in this case is the presence of congenital chylothorax—a rare but potentially life-threatening neonatal condition, with a reported incidence ranging from 1:240,000 to 1:5,775 in neonates (11, 12). Although this may be a coincidental finding, we hypothesize a potential developmental link. *ANKRD11* plays a vital role in chromatin remodeling and embryogenesis and may influence the development of the lymphatic system, including the thoracic duct. In other genetic syndromes, congenital chylothorax is a well-documented feature, typically due to lymphatic dysplasia. These examples highlight how early developmental gene dysregulation can manifest as structural lymphatic anomalies. While not currently recognized as a classical phenotype of KBG syndrome, congenital chylothorax may represent a rare and underreported component of its broader clinical spectrum, particularly in preterm infants or severe neonatal cases.

Even though there have been over 300 cases of KBG syndrome documented (7), the syndrome may be difficult to identify because many of the symptoms are mild and nonspecific (8, 13, 14). The diagnostic challenge of KBG syndrome is further amplified in the neonatal period, especially in preterm infants, where its variable and often subtle features may overlap with complications of prematurity, making early recognition particularly challenging. In our case, typical phenotypic features were initially absent, complicating clinical diagnosis and necessitating early molecular testing. As emphasized by Goldenberg et al. (3) and later studies (15), clinical evaluation alone is often insufficient; combining it with molecular testing, such as exome sequencing, is essential for accurate diagnosis. Our case exemplifies this, as early genetic testing was prompted by a constellation of severe early-onset features.

It was noteworthy that, upon reviewing the existing literature on KBG syndrome, we had not identified any studies specifically addressing preterm infants. The case mentioned in this report marks the first documented instance of a preterm infant diagnosed with KBG syndrome. Prematurity introduces additional complexity, as the immaturity of organ systems can exacerbate manifestations of underlying genetic conditions. In our patient, features such as congenital heart disease, chylothorax, and neurodevelopmental delays presented early and severely, potentially influenced by prematurity. Martinez-Cayuelas et al. (7) reported a 62.5% prevalence of cardiac anomalies in KBG syndrome. The presence of a large ventricular septal defect in our case aligns with these findings; however, in preterm infants, hemodynamic instability may occur more rapidly, necessitating earlier intervention. They require not only standard care for prematurity-related complications but also interventions tailored to the unique manifestations of KBG syndrome. Nutritional management played a pivotal role in the treatment of this patient. Initially, a specialized formula rich in medium-chain triglycerides (MCT) was used to minimize lymphatic flow and reduce chylous effusion, in accordance with established recommendations for managing infantile chylothorax. As the patient transitioned out of the acute phase, a high-caloric preterm formula was introduced to support catch-up growth and neurodevelopment. This strategy was guided by recommendations from neonatal nutrition guidelines and adjusted regularly by the multidisciplinary nutrition team. The dynamic adaptation of nutritional interventions reflects the need for individualized management plans in rare syndromic cases involving multiple organ systems. Considering the significant implications of prematurity on the management and prognosis of KBG syndrome patients, it is imperative for future research to explore the interplay between prematurity and KBG syndrome more thoroughly. Such studies should aim to enhance both the management practices and outcomes for this special population.

In terms of neurodevelopment, our patient showed motor and language delays, consistent with previous KBG syndrome reports such as Peluso et al. (16) MRI findings included bilateral ventricular enlargement, reduced hippocampal volume, and abnormal temporal lobe development—features that are suggestive of underlying structural brain involvement. Although specific imaging-genotype correlations for *ANKRD11* have not been fully established, *ANKRD11* is known to regulate neurodevelopment through chromatin remodeling. These imaging findings may therefore reflect downstream effects of disrupted gene function.

In conclusion, we report a premature newborn with KGB syndrome with a big ventricular septal defect and congenital chylothorax which expanded the phenotype of KBG syndrome which is easily misdiagnosed because of its unusual clinical profile and inadequate clinical knowledge of the condition. However, we also emphasized the importance of nutritional intervention in the treatment of this case, as well as the positive effects of interdisciplinary collaboration, which has improved the clinical outcomes of the patient.

## Data Availability

The datasets presented in this study can be found in online repositories. The names of the repository/repositories and accession number(s) can be found in the article/Supplementary Material.

## Supplementary material

Supplementary Figure S1 Prevalence of symptoms in KBG syndrome.
