# Supplementary figures and images for: A novel heterozygous mutation of ANKRD11 causes KBG syndrome in a preterm neonate: a case report and literature review
Source: Front Pediatr. 2025 Jun 12;13:1565261. doi: 10.3389/fped.2025.1565261 (PMC12198213; doi:10.3389/fped.2025.1565261)

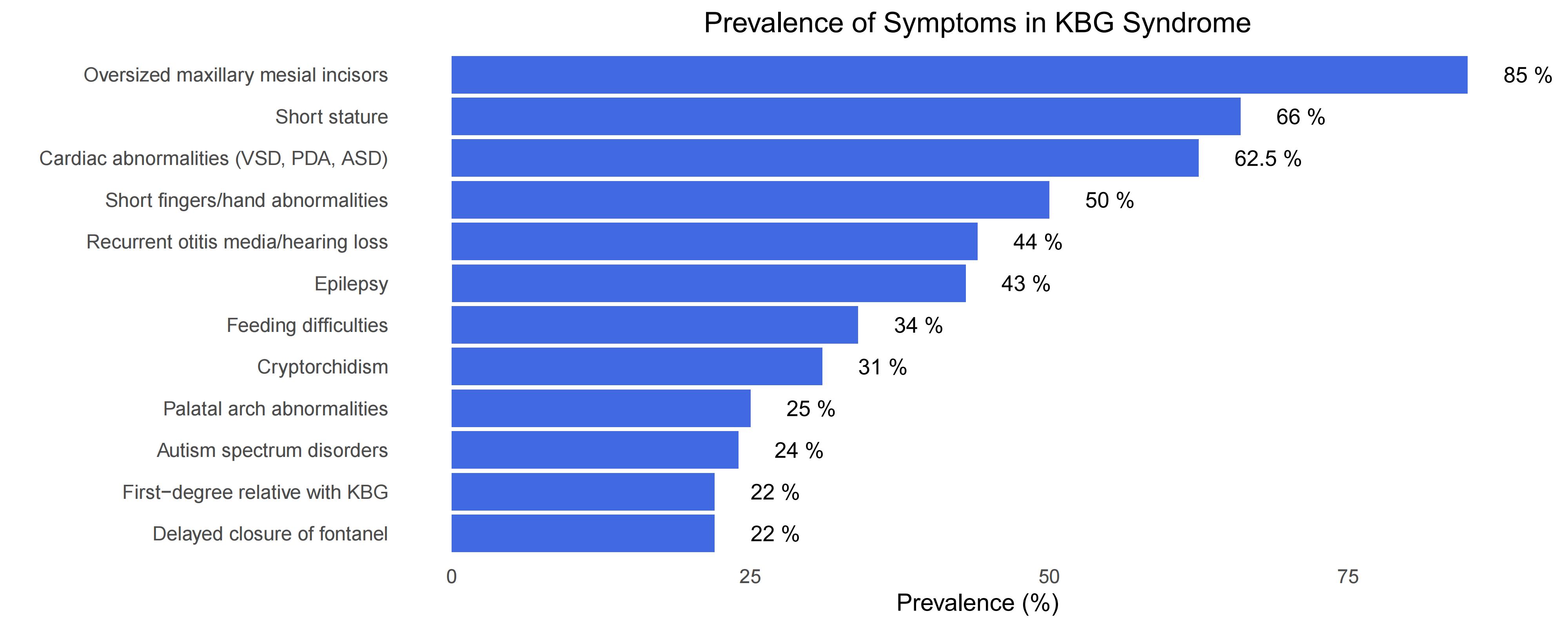

Supplement: Supplementary Figure S1 — Prevalence of symptoms in KBG syndrome. [file Image1.jpeg]
